# The Detection of Bovine Estrus by Lactoferrin Monoclonal Antibody

**DOI:** 10.3390/ani11061582

**Published:** 2021-05-28

**Authors:** Jihwan Lee, Suhyun Lee, Younbae Park, Seokhyun Lee, Seungmin Ha, Manhye Han, Gulwon Jang, Myunghum Park, Kyungwoon Kim, Hakjae Chung

**Affiliations:** 1 Dairy Science Division, National Institute of Animal Science, RDA, Cheonan-si 31000, Korea; leejh6735@korea.kr (J.L.); justusha@korea.kr (S.H.); hanmhh@korea.kr (M.H.); kwchang@korea.kr (G.J.); 2 Animal Breeding & Genetics Division, National Institute of Animal Science, RDA, Cheonan-si 31000, Korea; lhyungm@korea.kr; 3 MKbiotech, Daejeon 34134, Korea; pyb285@mkbiotech.co.kr; 4 Dairy Cattle Improvement Centre, Goyang-si 10292, Korea; asdko123@nate.com; 5 TNT Research, Jeonju-si 54810, Korea; pmh@tntresearch.co.kr; 6 Planning and Coordination Division, National Institute of Animal Science, RDA, Wanju-gun 55365, Korea; kw72kim@korea.kr; 7 Swine Science Division, National Institute of Animal Science, RDA, Cheonan-si 31000, Korea

**Keywords:** cattle, estrus, lactoferrin, monoclonal antibodies, heat detection kit

## Abstract

**Simple Summary:**

This study aimed to develop monoclonal antibodies with high specificity against bovine lactoferrin, which we have previously demonstrated to be overexpressed in bovine cervical mucus during estrus. Using an enzyme-linked immunosorbent assay, we observed that our monoclonal exhibited strong affinity for bovine lactoferrin protein. In addition, upon testing the new heat detection kits based on our antibody on 12 Korean native cows, we demonstrated an accurate detection of estrus during estrous synchronization. This is the first report of a non-invasive method to detect estrous using antibodies that bind to physiological material in cows. The results of this study suggest that the antibodies and a fabricated heat detection kit can be utilized to improve estrous detection in the cattle industry.

**Abstract:**

To improve reproductive performance in cattle, the accurate detection of estrus and optimization of insemination relative to ovulation are necessary. However, poor heat detection by farm staff leads to a decreased conception rate, thus inflicting economic damage to the beef and dairy industries. This study aimed to develop monoclonal antibodies (mAb) that can specifically bind to the bovine lactoferrin (bLF) protein, which we have previously demonstrated to be overexpressed in bovine cervical mucus during estrus. Female rats were intraperitoneally immunized with bLF protein as the antigen. Anti-bLF mAbs were then purified by affinity chromatography, and their binding affinity for the bLF antigen was examined using ELISA. We found a high binding affinity between mAbs and bLF. Finally, we developed a rapid bovine heat detection kit using the anti-bLF mAbs that we generated and tested on cervical mucus from 12 cows (estrous synchronization, *n* = 2; natural cycling, *n* = 10). We found that the kits accurately detected estrus. Overall, our fabricated heat detection kit based on rat anti-bLF mAbs could pave the way for the development of potent tools for heat detection devices for dairy cattle, thereby preventing economic loss.

## 1. Introduction

In South Korea, the milk production of dairy cows has tremendously increased over the past 30 years. Particularly, the 305-day adjusted milk yield of Holstein-Friesian cows increased by approximately 40% from 6176 kg in 1990 to 10,352 kg in 2019 through the government’s herd improvement program [1]. The increase in milk yield leads to decreased fertility, which induces anestrus, silent heat, and anovulation in dairy herds [2,3,4]. An irregular estrous cycle also interferes with the optimal period of insemination, resulting in repeat breeders [5,6], which can cause significant economic losses in the cattle industry. Esslemont (1997 yr) and Senger (1994 yr) reported that the economic loss caused by poor heat detection in the UK and US was estimated to be £200 million and $300 million per year, respectively [7,8,9]. According to data released by Statistics Korea (2020), misdiagnosis or failure to detect estrus results in approximately $378 per cow during one estrous cycle (21 days) (http://kostat.go.kr/portal/eng/index.action, accessed on 5 January 2021). A recent survey on the reasons for involuntary culling among dairy farmers in South Korea revealed that reproductive failure accounted for the highest proportion (31.4%) [10].

The optimal timing of artificial insemination via accurate estrus detection is also important to improve cow fertility. Dairy farmers have primarily relied on visual observation and heat mount detectors (KaMar, Scratchcard) for estrus detection. Recently, the use of automated activity monitors (AAM), such as neck collars and pedometers, has been increasing. However, because AAM devices depend on increased activity during estrus, they have a low detection rate in cows with a high milk yield, a low body condition score (<2), or lameness. These animals do not exhibit estrous behavioral signs [11].

Previously, we had investigated differential expression of proteins in the cervical mucus during estrus using SELDI-TOF MS, SDS- PAGE, MALDI-TOF/TOF, and RT-qPCR and revealed that the bovine lactoferrin (bLF) protein is a biomarker of estrus [12]. We focused on this specific protein secreted by female reproductive organs during estrus in cattle. We hypothesized that detection of this protein could accurately ascertain estrus in animals that do not exhibit estrous behavioral signs. Commercially available LF antibodies are mostly produced based on human amino acid sequences obtained from breast milk samples. However, to the best of our knowledge, there are few commercially available anti-bLF monoclonal antibodies, and in addition, most manufacturers do not specify the epitope information of the antibodies. Moreover, the small number of known epitopes targetted by commercial antibodies are different from those predicted in this study. Furthermore, to the best of our knowlege, the use of mAbs of bLF for detection of estrus has not yet been investigated. Therefore, we conducted this study on the development of anti-bLF mAbs.

In this study, we also aimed to develop a heat detection kit based on the anti-bLF mAb that specifically binds to bLF, present in the cervical mucus. The use of body fluid for estrus detection is particularly important in animals in silent-heat, even though the female reproductive tracks undergo a normal estrous cycle. Our study can improve fertility by detecting estrus in animals that do not exhibit estrous signs, which will contribute to economic benefits to the dairy and beef industry by reducing the days open and in calving intervals.

## 2. Materials and Methods

### 2.1. Experimental Animals

Female Wistar–Imamichi rats (*n* = 5, 8- to 10-week old) purchased from Japan SLC (Shizuoka, Japan) were housed in specific pathogen-free conditions for immunization. They were maintained under a 12:12 light/dark cycle, and fed with commercial rat chow (Purina, Seoul, South Korea) and tap water ad libitum. All animal experimental procedures were approved by the National Institute of Animal Science Animal Care and Ethics Committee in South Korea (approval number NIAS-109).

### 2.2. Peptide Conjugation

Peptide synthesis was carried out to produce bLF mAbs that specifically recognize post-translational modifications (phosphorylation and protease degradation) using 5 repetitive synthesis steps. The amino acid sequence of *Bos taurus* lactoferrin containing 708 amino acids (NP_851341.1) was obtained from the National Center for Biotechnology Information (NCBI).

bLF antigen regions were carefully predicted based on antigenicity and hydrophobicity of the whole amino acid sequence using the online software ProtScale at https://web.expasy.org/protscale/ (accessed on 18 May 2017) (ExPASy, Switzerland) [13,14,15]. A 16-mer peptide of KAQEKFGKNKSRSFQ from bovine lactoferrin corresponding to amino acids 292–306 was selected as the immunogen.

To facilitate conjugation to carrier proteins, a cysteine residue was added to the end of the C-terminus of the individual peptides. Keyhole Limpet Hemocyanin (KLH)-conjugated peptides were used as carrier molecules for generating the rat monoclonal antibody. All procedures were carried out according to the manufacturer’s instructions on the conjugation kit (Sigma-Aldrich, St. Louis, MO, USA). Briefly, 5 mg of KLH was dissolved in 0.5 mL Phosphate-buffered saline (PBS), 3 mg of Maleimidobenzoic acid-N-hydroxysuccinimide ester (MBS) was dissolved in 200 μL Dimethyl Formamide (DMF). Then, to make carrier protein conjugated peptides, 70 μL of MBS solution was added to the 0.5 ml KLH solution. After stirring, the mixture was incubated for 30 min at room temperature. The KLH/MBS mixture passed through a PD-10 desalting column overnight using PBS. The purified KLH/MBS mixture was collected and 0.5 mL of deionized water was added. Next, 5 mg of peptide was dissolved in 100 μL of DMF. Then, 1 mL of purified KLH/MBS mixture was added to the peptide solution and shaken rapidly. Next, 2N NaOH (11 μL) was added and the solution was incubated overnight at 4 °C. The next day, finally 3 mL of ammonium biocarbonate was added to mixture before lyophilizing the reaction solution. The carrier protein conjugated peptide solution was stored at −20 °C until later use.

### 2.3. Production of Rat Anti-bLF mAbs

Female rats were intraperitoneally immunized four times (the control animal was injected with only PBS). Conjugated KLH-peptide (100 μg) was mixed with complete Freund’s adjuvant (100 μg) for the first injection. The final volume did not exceed 100 μL. For the second and third booster injections, 50 μg of peptide-KLH with incomplete Freund’s adjuvant (Sigma-Aldrich, St. Louis, MO, USA) were injected after a two-week interval. The last booster injection contained phosphate-buffered saline (PBS, 50 μL) (without any adjuvant), 6 weeks after the initial injection. To measure the antibody titer, blood serum was obtained from the rat tail vein, and a serum enzyme-linked immunosorbent assay (ELISA) was performed using a microplate reader (Bio-Rad model 450). The wells of the ELISA micrometer plate (Nunc, Roskilde, Denmark) were coated with 50 μL of bLF extracted from bovine cervical mucus (5 μL/mL in PBS) and incubated for 2 h at 37 °C. After the coating solution was discarded, the plates were washed twice with PBS and blocked with 1% Bovine Serum Albumin (BSA) and 0.1% Tween 20 in PBS for 1 h at 37 °C. Wells were washed twice with PBS once again. Then, the rat sera (50 μL) was added to each well in 10-fold serial dilutions starting from 1:10. The plate was incubated for 2 h at 37 °C and then washed thrice with PBS. Horseradish peroxidase (HRP)-conjugated rabbit anti-rat IgG (Razi Biotech, Gatley, UK) was added to each well (50 μL/well; 1:5000 dilution) and incubated for 1 h at 37 °C. The plates were washed five times, and the Tetramethylbenzidine (TMB) subtrate (50 μL; color reagents) was added to each well in the dark for 1 h at room temperature (25 ± 1 °C). The reaction was quenched with 1 N H_2_SO_4_ solution, and an optical density (OD) value at 450 nm was recorded using a microplate reader (BioRad, Hercules, CA, USA). Rats with a higher titer of antibody were selected for fusion.

Three days after the last booster injection, the rat was euthanized, and cells from the spleen were harvested. The cells (1 × 10^8^) were washed with serum-free Dulbecco’s modified eagle medium (Gibco, Thermo Fisher Scientific, Carlsbad, CA, USA) containing myeloma cells (2 × 10^7^, Sp2/O-Ag14 cell line) in a 5:1 ratio diluted using polyethylene glycol 4000 for fusion. The fused cells were cultured in four 96-well plates (Becton Dickinson Labware, Bedford, MA, USA) with the hypoxanthine-aminopterin-thymidine (HAT) selection medium (GIT medium, Wako Pure Chemical Industries, Osaka, Japan) containing 10% fetal bovine serum and incubated at 37 °C with 5% CO_2_. Four days later, the HAT selection medium was added to each well, and the culture medium was freshly replaced every four days. Colony formations were monitored daily. When the colony diameter reached 1 mm, the presence of antibody was determined by ELISA. Twelve days after fusion, the supernatants were collected for further analysis.

### 2.4. Assesement of Rat Anti-bLF mAbs

The supernatants were screened for the production of anti-bLF mAbs using ELISA. A 96-well ELISA micrometer plate (Nunc, Roskilde, Denmark) was coated with 50 μL of bLF extracted from bovine cervical mucus [12] (5 μg/mL in PBS) and incubated for 2 h at 37 °C. The coating solution was discarded, and the plates were washed twice with PBS and blocked with 1% BSA in 0.1% Tween 20 in PBS for 1 h at 37 ℃. The anti-bLF (50 μL) was diluted in series of 1:10, 1:10^2^, 1:10^3^, 1:10^4^ and 1:10^5^. The plates were washed with PBS, and the supernatant (anti-bLF mAbs) was added to each well and incubated for 2 h at 37 °C. The plates were washed thrice with PBS, and horseradish peroxidase (HRP)-conjugated rabbit anti-rat IgG (Razi Biotech, Gatley, UK) was added to each well (50 μL/well; 1:5000 dilution) and incubated for 1 h at 37 °C. Then, the plates were washed five times, and the TMB substrate solution (50 μL; color reagents) was added to each well in the dark for 1 h at room temperature (25 ± 1 °C). The reaction was quenched with 1 N H_2_SO_4_ solution, and the OD value at 450 nm was recorded using a microplate reader (BioRad, Hercules, CA, USA).

### 2.5. Evaluation of Heat Detection Kit

**Figure 1:**
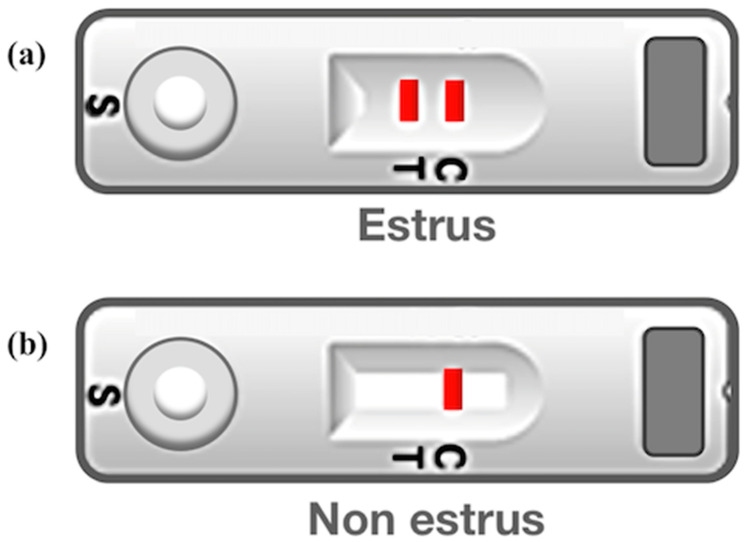
Results of fabricated bovine heat detection kit. (**a**) A positive test result for estrus is indicated by two red lines. (**b**) A negative test result for estrus (i.e., non-estrus) is indicated by one red line. C, control line; T, Test line; S, sample (the location to drop the sample).

We have previously registered a patent for bLF found in the cervical mucus of cows at estrus at the Korean Intellectual Property Office [16]. The bovine heat detection kit was manufactured by a Korean company (Cat. BHD10-101, TNT research, Jeonju-si, Korea) through joint research with our institute. We evaluated the kit we developed based on anti-bLF mAbs in Korean native cattle (Hanwoo) raised in our laboratory center. Twelve cows were randomly selected and divided intro three groups: Group 1 was subjected to estrous synchronization using the CIDR-Ovsynch method (*n* = 2); Group 2 exhibited standing-to-be-mounted behaviors (*n* = 8); and Group 3 exhibited a corpus leuteum more than 20 mm in diameter indicating non-estrus (*n* = 2). For Group 1, cervical mucus was collected by swabbing at the following periods: 24 h before the second Gonadotropin-releasing hormone (GnRH) administration (−24 h); at the time of the second GnRH administration (0 h); and 8, 16 and 48 h (0 h, 8 h, 16 h, 48 h) after the second GnRH administration. We inserted a swab approximately 10 cm into the cow vagina and gently rotated it 4–5 times so that a sufficient amount of mucus was obtained. The swabs were then placed in tubes containing PBS (2 mL) and shaken five times. Five to seven drops (10 μL/drop) of the mucus solution were dotted to the circular specimen spot of the device to react with the antibody (sandwich ELISA method). Changes were observed for 5 min. When cows were in oestrus, the device would display two red lines; otherwise, only one line would appear. The color change in the second line is dependent on the intensity of estrus (Figure 1).

### 2.6. Statistical Analysis

All data were expressed as the means with standard error (SE) and were visualized in R 3.1.2 [17].

## 3. Results

### 3.1. Prediction of Antigenic Peptides for bLF Amino Acid Sequence

We found seven predicted candidate antigenic peptide regions from bLF amino acid sequences (Table 1). Among them, the most suitable was prediction #3 from bLF corresponding to amino acids 292–306 (KAQEKFGKNKSRSFQ, 16-mer), which was then selected as immunogens. The rat serum antibody titers were analyzed by ELISA (Table 2).

We aligned our predicted immunogen sequence (Prediction #3) with the sheep (NP_001020033.1), human (NP_002334.2), mouse (NP_032548.2) and pig (NP_999527.2) lactoferrin sequence to find out whether the similar sites exist using the online software Cluster Omega (1.2.4) (http://www.ebi.ac.uk/Tools/msa/clustalo/, accessed on 20 January 2021). The multiple sequence alignment revealed that seven amino acids were conserved across species.

### 3.2. Production of Rat Anti-bLF mAbs

Rat anti-bLF monoclonal antibodies were obtained from hybridomas. Among them, clone 4 had the highest binding affinity according to the ELISA results and was thus further analyzed (Table 3).

### 3.3. Determination of Binding Affinity by ELISA

**Figure 2:**
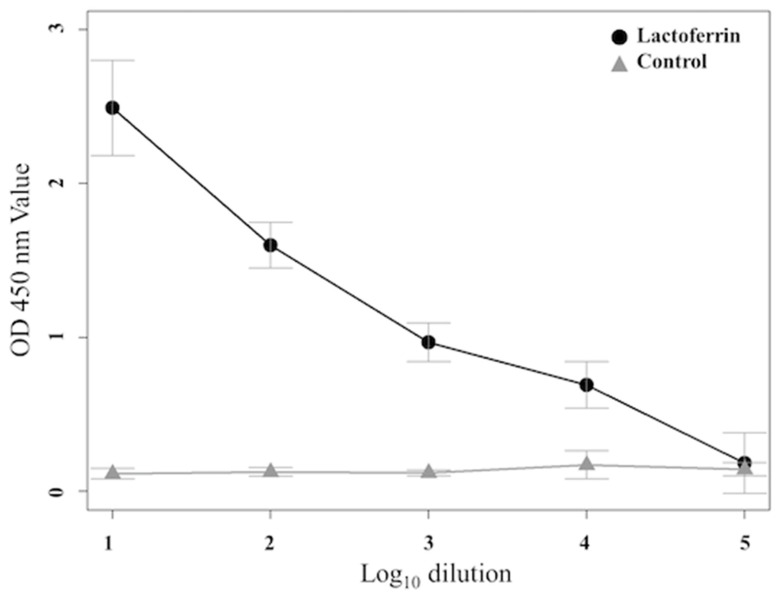
Sensitivity testing results for bLF antigen detection using ELISA. OD values at 450 nm of are presented as the mean ± SE under 1:10 to 1:10^5^ dilution ratio.

The binding affinity (OD value) of the rat anti-bLF mAbs to the antigen was analyzed using ELISA at 450 nm. We assessed the binding affinity of the bLF to the mAb at 1:10–1:10^5^ dilution of mAb (Figure 2). Increasing mAb concentration corresponded to an increased binding affinity. At 450 nm, the binding affinity values of anti-bLF mAbs were significantly higher than that of the control at 1:10 to 1:10^4^ dilutions, but not at 1:10^5^. The same results were obtained in three biological replicates.

### 3.4. Estrous Discrimination Using Bovine Heat Detection Kit

**Figure 3:**
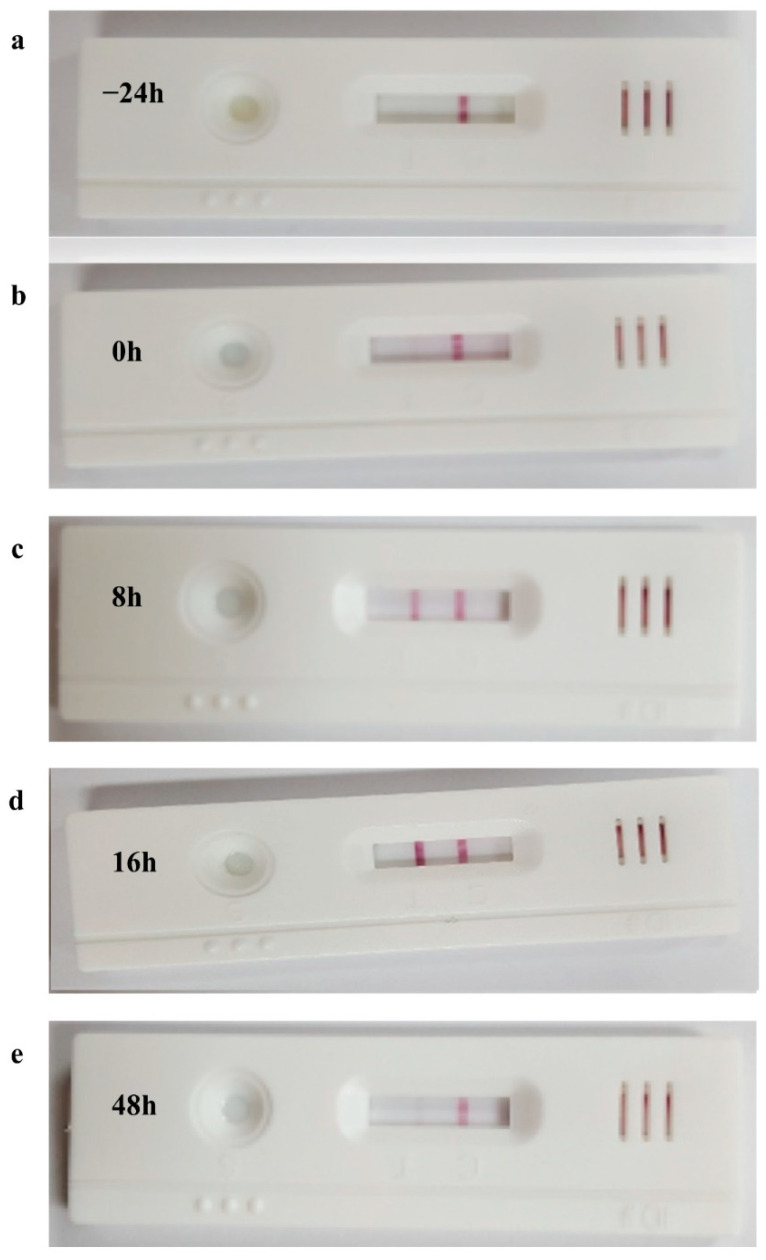
Estrous discrimination using bovine heat detection kit during estrous synchronization, (**a**) 24 h before the second GnRH injection; (**b**) at the time of the second GnRH injection; (**c**–**e**) at 8, 16, 48 h after the second GnRH injection, respectively.

**Figure 4:**
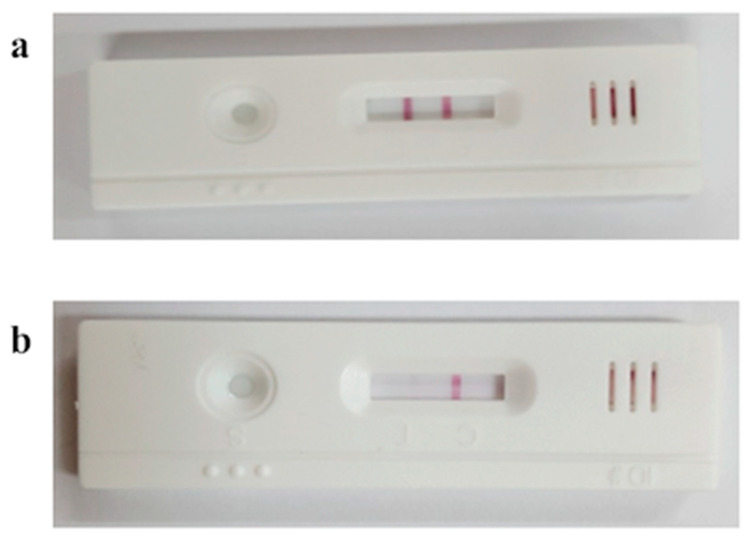
Estrous discrimination using bovine heat detection kit during estrus (**a**, Group 2) and non-estrus (**b**, Group 3).

To test whether this kit accurately detects estrus, we divided the cows into three groups and diagnosed estrus using bovine cervical mucus in each animal. In the case of the estrous-synchronized Group 1, cervical mucus was collected and reacted to the heat detection kit at −24 h, 0 h, 8 h, 16 h, and 48 h from the time of the second GnRH administration (CIDR-Ovsynch method). Although only one line was observed at −24 h (i.e., non-estrus), a faint second line gradually began to appear from 0 h (i.e., early estrus), and the second red line was clearly visible at 8 h and 16 h (i.e., estrus; may be optimal insemination time), and again only one line was observed at 48 h (i.e., non-estrus) (Figure 3). In the case of Group 2 (standing to be mounted under natural estrous cycle) cows, the second red line (indicating estrus) was clearly identified for all cows (Figure 4). In Group 3 (corpus luteum diameter ≥ 20), only one red line (i.e., non-estrus) was observed (Figure 4). All raw figures for Groups 1–3 were presented in Supplementary Figures S1–S3.

## 4. Discussion

Artificially inseminating cattle at the optimal time after accurate estrous will enhance the conception rate in cattle [18]. The duration of estrus in dairy cows has been reduced by approximately 20.6% in recent years (from 18 h to 14.3 h) compared with that in the past 70 years [19,20,21]. The ratio of silent heat was diagnosed from approximately 10 to 40% in dairy herds [22,23,24]. It is difficult to accurately detect estrus based on animal behaviors and visual observation alone. In addition, several factors, such as the floor-surface condition of the barn [25], climate (heat stress condition) [26], lameness, body condition score, and other physiological and genetic factors (high yielding cows), affect estrus detection [11]. Although several studies have been conducted on bovine estrus detection, non-invasive and accurate methods for estrus detection using specific proteins secreted by the cervical mucus have remained elusive. This study focused on the cervical mucus, whose production is induced during estrus in cattle. Previously, we have shown that bLF expression is significantly enhanced (more than two-fold) in bovine cervical mucus during estrus, as compared to non-estrus cattle [12].

In this study, we developed highly specific rat anti-bLF monoclonal antibodies that exhibited a high binding affinity. Using these antibodies, we then developed a bovine heat detection kit and achieved accurate detection results of estrus in both estrous synchronized and natural cycling cows. Furthermore, the color change of the second red line was shown to be dependent on the intensity of estrus, which will help in determining the optimal time for insemination.

The detection kit we developed has been tested in a previous study on five farms raising Korean native cattle and yielded a 64% conception rate [27]. This rate is similar to the average conception rate of Korean native cattle raised in farms with good estrous detection rates [28]. Our detection kit is available to the dairy and beef industry for estrous detection. Furthermore, it is useful to non-expert users as it involves a simple, non-invasive protocol to collect samples (cervical mucus). However, in future studies, we need to test this kit for many experimental animals to find the errors and increase the accuracy of estrous diagnosis.

Lactoferrin(LF) is among the major estrogen-inducible proteins [29,30] present in many biological fluids [31], such as saliva, tears, and semen. It can kill bacteria and viruses and regulate inflammatory and immune responses [32]. Increased estrogen levels in cattle induces estrus and increases LF levels, which may trigger an immune response to bacteria and viruses that enter cattle via dilated cervical orifices during estrus [29,30,32].

**Figure 5:**
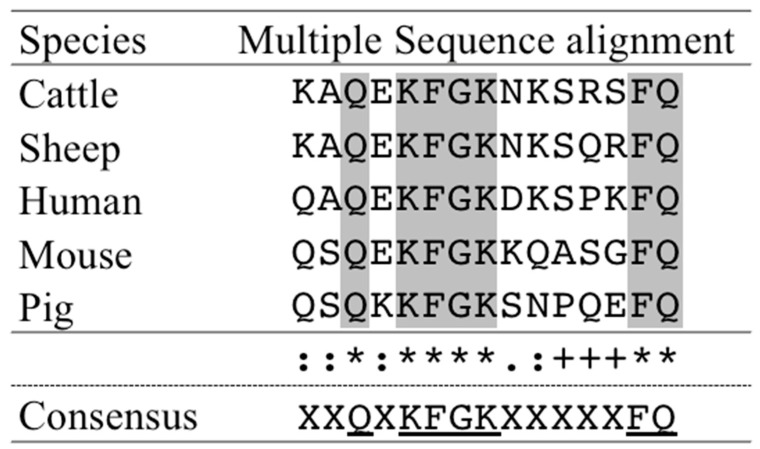
The sequence of cattle lactoferrin immunogen peptide was multiple aligned with the corresponding regions of the lactoferrin peptide from other species. The consensus amino acids are underlined and are highlighted in gray. (*) indicates fully conserved residue. (:) indicates conserved amino acid substitutions. (.) indicates semi-conserved substitution. (+) indicates no conservation.

Multiple sequence alignment was performed using lactodferrin sequences from sheep (NP_001020033.1), human (NP_002334.2), mouse (NP_032548.2), and pig (NP_999527.2) and bovine lactoferrin. We found that the LF of other species could also be recognized by this anti-bLF monoclonal antibody, because we used conserved regions, and seven amino acids (XXQXKFGKXXXXXFQ), as antigens. Glutamine Q (3, 15), lysine K (5, 8), Phenylalanine F (6, 14), Glycine G (7) might represent the potential epitope recognized by our lactoferrin monoclonal antibody (Figure 5). However, further studies are needed to confirm this hypothesis. This antibody could be used as a powerful marker in estrus detection in domesticated animals.

In the present study, we developed a mAb against bLF. We also developed a heat detection kit and tested it on randomly selected cows. To the best our knowlege, this is the first estrus detection method based on a mAb against the bLF protein. However, our small sample size may be a limiting factor in determing the accuracy, specificity, and sensitivity of this kit. In addition, our results must be validated in repeat breeders, cattle in silent heat and high yielding cows in the future.

In summary, we established an accurate estrus detection kit for cattles using bLF mAb. Our findings contribute to the improvement of conception rate in dairy cattle and could provide economic benefits to farmers by reducing open days and calving intervals.

## 5. Conclusions

A previous study revealed that lactoferrin was a biomarker for bovine estrus. In this study, we developed monoclonal antibodies that can bind to bLF and developed a heat detection kit using these monoclonal antibodies. Our tests revealed that the heat detection kits accurately detected lactoferrin from bovine cervical mucus collected during estrus. Our research may contribute to economic benefits by improving detection of fertility in cattle that do not show estrous signs.

## Figures and Tables

**Table 1:** Prediction of protein antigenic determinants based on antigenicity and hydrophobicity from bLF amino acid sequences.

Prediction #	Location	Amino Acid Sequence
1	64–77 amino acid	CIRAIAEKKADAVT
2	234–247 amino acid	FENLPEKADRDQYE
3	292–306 amino acid	KAQEKFGKNKSRSFQ
4	348–363 amino acid	KNLRETAEEVKARYTR
5	431–444 amino acid	AENRKSSKHSSLDC
6	574–588 amino acid	ESTADWAKNLNREDF
7	649–664 amino acid	CLFKSETKNLLFNDNT

The ‘#’ symbol stands for number.

**Table 2:** Serum of the rat immunized with bLF was assayed by ELISA at OD 450 nm (mean ± SE).

Rat Serum	Control	1:10	1:10^2^	1:10^3^	1:10^4^	1:10^5^
1	0.17 ± 0.07	2.55 ± 0.04	1.88 ± 0.06	0.94 ± 0.10	0.64 ± 0.09	0.21 ± 0.06
2	0.17 ± 0.04	2.79 ± 0.07	2.00 ± 0.07	1.12 ± 0.06	0.74 ± 0.07	0.28 ± 0.06
3	0.19 ± 0.08	3.01 ± 0.14	1.67 ± 0.09	0.96 ± 0.08	0.67 ± 0.11	0.22 ± 0.07
4	0.14 ± 0.07	2.31 ± 0.06	1.52 ± 0.06	0.84 ± 0.08	0.60 ± 0.08	0.19 ± 0.05

**Table 3:** Screening of rat anti-bLF mAb based on binding affinity (OD) at OD = 450 nm using ELISA.

Binding Affinity (Mean ± SE)	Control	Clone 1	Clone 2	Clone 3	Clone 4
mAb-bLF	0.12 ± 0.02	0.86 ± 0.11	2.28 ± 0.07	2.07 ± 0.18	2.49 ± 0.15

## Data Availability

The data presented in this study are available on request from the corresponding author.

## Supplementary Materials

The following are available online at https://www.mdpi.com/article/10.3390/ani11061582/s1, Figure S1: Estrous discrimination using bovine heat detection kit during estrous synchronization (*n* = 2, Group 1), Figure S2: Estrous discrimination using bovine heat detection kit during estrus (*n* = 8, Group 2), Figure S3: Estrous discrimination using bovine heat detection kit during non-estrus (*n* = 2, Group 3).

## References

[1] Seo M.D.H.I. Annual Report in Korea. http://www.dcic.co.kr/_uploadFiles/_annual_report/2019_main.pdf.

[2] Walsh S., Williams E., Evans A. (2011). A review of the causes of poor fertility in high milk producing dairy cows. Anim. Reprod. Sci..

[3] Albarran-Portillo B., Pollott G. (2013). The relationship between fertility and lactation characteristics in Holstein cows on United Kingdom commercial dairy farms. J. Dairy Sci..

[4] Zduńczyk S., Janowski T., Raś M. (2005). Current Views on the Phenomenon of Silent Heat in Cows. Med. Weter..

[5] Roelofs J.B., van Erp-van der Kooij E. (2018). Estrus Detection Tools and Their Applicability in Cattle: Recent and Perspectival Situation. Anim. Reprod..

[6] Yusuf M., Nakao T., Ranasinghe R.B.K., Gautam G., Long S.T., Yoshida C., Koike K., Hayashi A. (2010). Reproductive performance of repeat breeders in dairy herds. Theriogenology.

[7] Garforth C., McKemey K., Rehman T., Tranter R., Cooke R., Park J., Dorward P., Yates C. (2006). Farmers’ attitudes towards techniques for improving oestrus detection in dairy herds in South West England. Livest. Sci..

[8] Esslemont R.J., Kossaibati M.A. (1997). Culling in 50 dairy herds in England. Vet. Rec..

[9] Senger P. (1994). The Estrus Detection Problem: New Concepts, Technologies, and Possibilities. J. Dairy Sci..

[10] Baek K.S., Lee W.S., Park S.J., Lim H.J., Son J.K., Kim S.B., Kwon E.G., Jung Y.S., Kim K.H. (2011). The Accuracy Analysis and Applied Field Research of a Newly Developed Automatic Heat Detector in Dairy Cow. Reprod. Dev. Biol..

[11] Holman A., Thompson J., Routly J.E., Cameron J., Jones D.N., Grove-White D., Smith R., Dobson H. (2011). Comparison of oestrus detection methods in dairy cattle. Vet. Rec..

[12] Lee W., Park M., Kim K., Song H., Kim K., Lee C., Kim N., Park J., Yang B., Oh K. (2016). Identification of lactoferrin and glutamate receptor-interacting protein 1 in bovine cervical mucus: A putative marker for oestrous detection. Reprod. Domest. Anim..

[13] Kolaskar A., Tongaonkar P.C. (1990). A semi-empirical method for prediction of antigenic determinants on protein antigens. FEBS Lett..

[14] Hopp T.P., Woods K.R. (1981). Prediction of protein antigenic determinants from amino acid sequences. Proc. Natl. Acad. Sci. USA.

[15] Mishra S., Gomase V.S. (2016). Identification of Antigenic Determinants, Solvent Accessibility and MHC Binders of Antigen NADH Dehydrogenase Subunit 5 from a Little Dragon from Medina (Dracunculusmedinensis). Drug Des..

[16] Chung H.J., Kim G.W., Park J.G., Yang B.C., Han D.W., Lee M.S., Lee S.D., Hong S.K., Chang W.K. (2012). Composition, Kit and Method for Determining Optimal Insemination Time of Hanwoo. KR Patent.

[17] RStudio Team (2019). RStudio: Integrated Development for R.

[18] Dransfield M., Nebel R., Pearson R., Warnick L. (1998). Timing of Insemination for Dairy Cows Identified in Estrus by a Radiotelemetric Estrus Detection System. J. Dairy Sci..

[19] Trimberger G.W. (1948). Breeding Efficiency in Dairy Cattle from Artificial Insemination at Various Intervals before and after Ovulation. Biology.

[20] Silper B., Madureira A., Kaur M., Burnett T., Cerri R. (2015). Short communication: Comparison of estrus characteristics in Holstein heifers by 2 activity monitoring systems. J. Dairy Sci..

[21] Reith S., Hoy S. (2018). Review: Behavioral signs of estrus and the potential of fully automated systems for detection of estrus in dairy cattle. Animal.

[22] Zduńczyk S., Janowski T., Raś A., Barański W. (2009). Accuracy of Ultrasonography and Rectal Palpation in the Diagnosis of Silent Heat in Cows Compared to Plasma Progesterone Concentration. Bull. Vet. Inst. Pulawy.

[23] Oltenacu P.A., Hultgren J., Algers B. (1998). Associations between use of electric cow-trainers and clinical diseases, reproductive performance and culling in Swedish dairy cattle. Prev. Vet. Med..

[24] Zduńczyk S., Mwaanga E.S., Małecki-Tepicht J., Barański W., Janowski T. (2002). Plasma Progesterone Levels and Clinical Findings in Dairy Cows with Post-Partum Anoestrus. Bull. Vet. Inst. Pulawy.

[25] Platz S., Ahrens F., Bendel J., Meyer H., Erhard M. (2008). What Happens with Cow Behavior When Replacing Concrete Slatted Floor by Rubber Coating: A Case Study. J. Dairy Sci..

[26] Gangwar P., Branton C., Evans D. (1965). Reproductive and Physiological Responses of Holstein Heifers to Controlled and Natural Climatic Conditions. J. Dairy Sci..

[27] Choi S.-H., Jin H.-J. (2017). Effects of Optimal Heat Detection Kit on Fertility after Artificial Insemination (AI) in Hanwoo (Korean Native cattle). J. Anim. Reprod. Biotechnol..

[28] Lee M.-S., Rahman S., Kwon W.-S., Chung H.-J., Yang B.-S., Pang M.-G. (2013). Efficacy of four synchronization protocols on the estrus behavior and conception in native Korean cattle (Hanwoo). Theriogenology.

[29] Pentecost B., Teng C. (1987). Lactotransferrin is the major estrogen inducible protein of mouse uterine secretions. J. Biol. Chem..

[30] Teng C.T. (1999). Regulation of Lactoferrin Gene Expression by Estrogen and Epidermal Growth Factor: Molecular Mechanism. Cell Biochem. Biophys..

[31] Teng C.T. (2002). Lactoferrin gene expression and regulation: An overview. Biochem. Cell Biol..

[32] González-Chávez S.A., Arévalo-Gallegos S., Rascón-Cruz Q. (2009). Lactoferrin: Structure, function and applications. Int. J. Antimicrob. Agents.

