# Supplementary material for: The Detection of Bovine Estrus by Lactoferrin Monoclonal Antibody
Source: Animals (Basel). 2021 May 28;11(6):1582. doi: 10.3390/ani11061582 (PMC8228451; doi:10.3390/ani11061582)
Supplement: Supplementary file 1 [file animals-11-01582-s001.zip › animals-1235275-supplementary/animals-1235275-supplementary.pptx]

## Slide 1
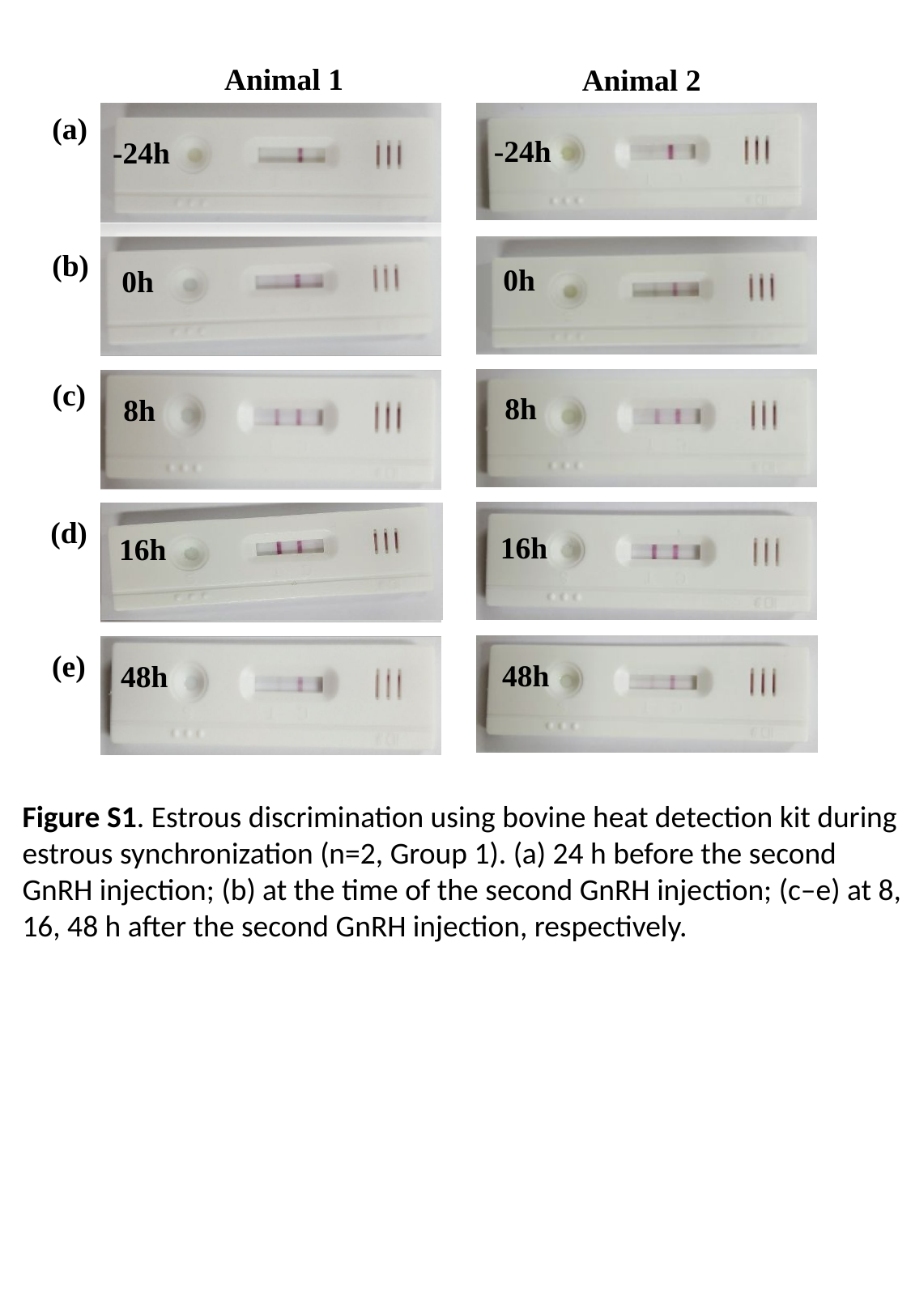

Animal 1
Animal 2
(a)
-24h
-24h
(b)
0h
0h
(c)
8h
8h
(d)
16h
16h
(e)
48h
48h
Figure S1. Estrous discrimination using bovine heat detection kit during estrous synchronization (n=2, Group 1). (a) 24 h before the second GnRH injection; (b) at the time of the second GnRH injection; (c–e) at 8, 16, 48 h after the second GnRH injection, respectively.

## Slide 2
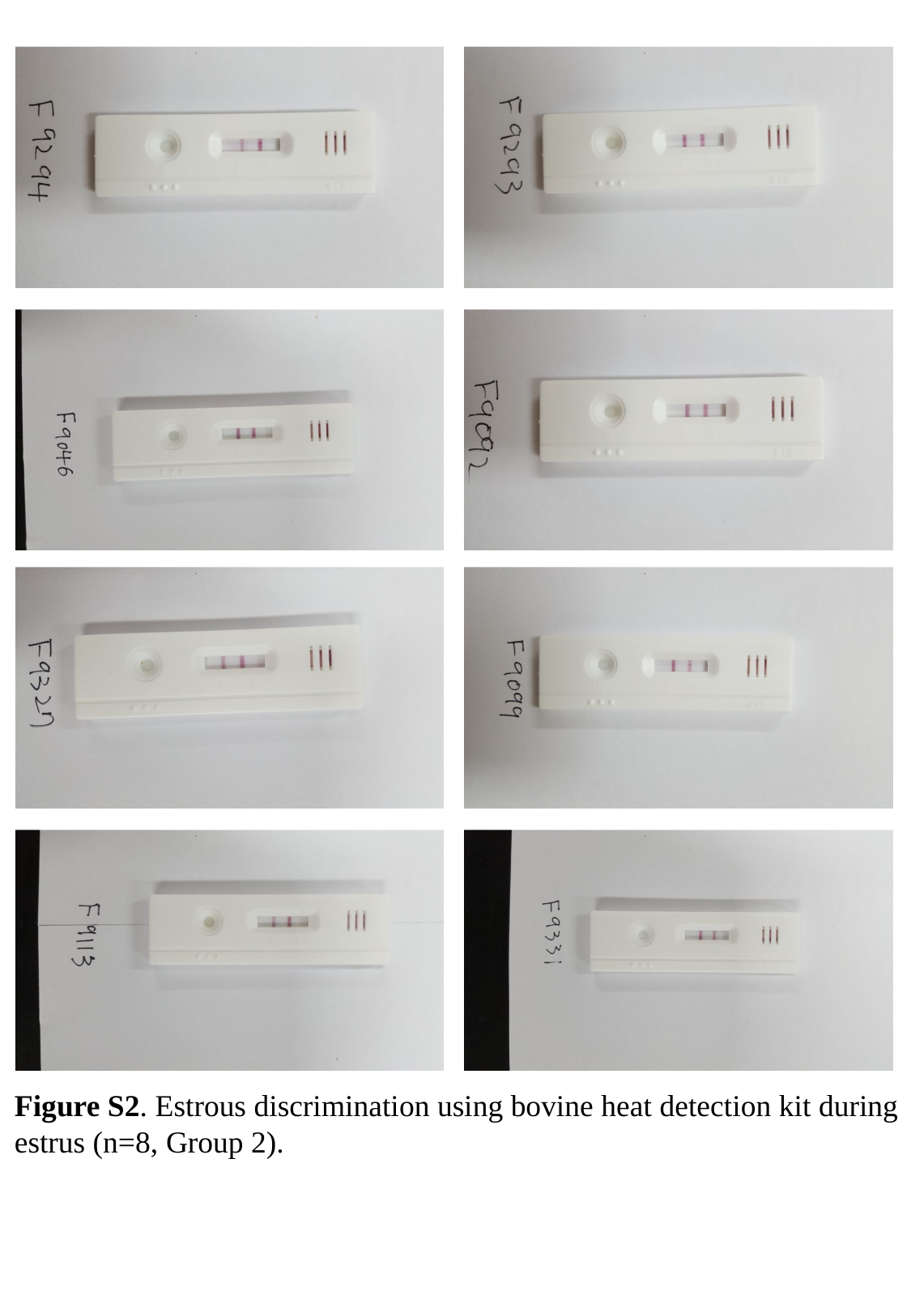

Figure S2. Estrous discrimination using bovine heat detection kit during estrus (n=8, Group 2).

## Slide 3
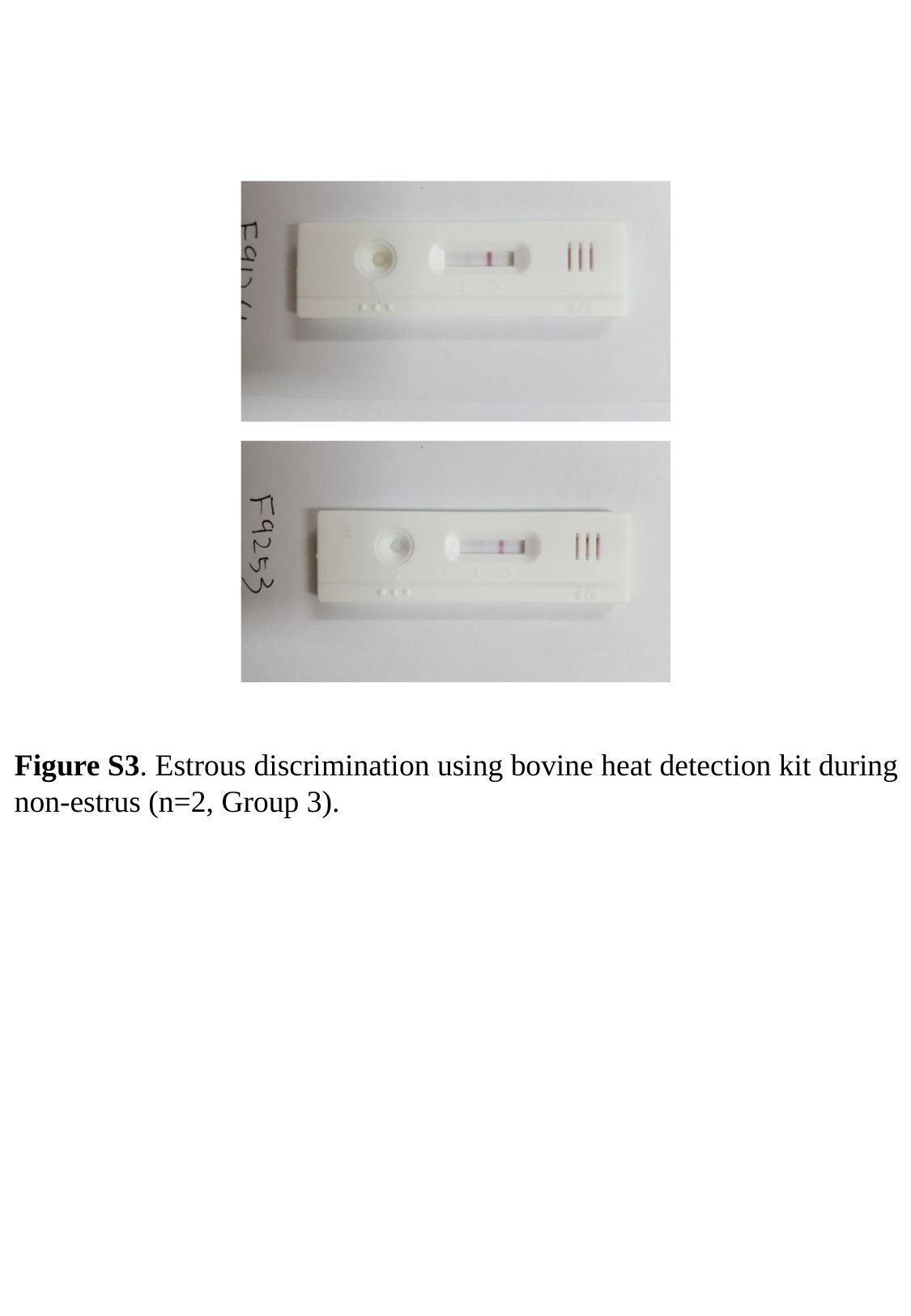

Figure S3. Estrous discrimination using bovine heat detection kit during non-estrus (n=2, Group 3).
